# Left ventricular contractile performance and heart failure in patients with left ventricular ejection fraction more than 40%

**DOI:** 10.1007/s00380-020-01641-w

**Published:** 2020-06-05

**Authors:** Shuichi Kitada, Yu Kawada, Satoshi Osaga, Marina Kato, Shohei Kikuchi, Kazuaki Wakami, Yoshihiro Seo, Nobuyuki Ohte

**Affiliations:** 1 Department of Cardiology, Nagoya City University, 1 Kawasumi, Mizuho-cho, Mizuho-Ku, Nagoya, Japan; 2 Department of Medical Innovation, Nagoya City University, Nagoya, Japan

**Keywords:** Systolic dysfunction, Heart failure, Mid-range LVEF, Inertia force of late-systolic aortic flow

## Abstract

Heart failure (HF) with mid-range left ventricular ejection fraction (LVEF) (HFmrEF) is considered a new category of HF and LVEF < 50%, which is the upper threshold of LVEF for HFmrEF, is thought to represent a mild decrease in LV contractile performance. We aimed to consider an LVEF threshold value to be taken as a surrogate for impairment of LV contractile performance, resulting in new-onset HF. We enrolled 398 patients with LVEF ≥ 40% that underwent cardiac catheterization. Using the LV pressure recording with a catheter-tipped micromanometer, we calculated the inertia force of late systolic aortic flow (IFLSAF), which was sensitive to the slight impairment in LV contractile performance. We evaluated the utility of the IFLSAF for predicting future cardiovascular death or hospitalization for HF. We performed a receiver operating characteristic (ROC) curve analysis to determine the best LVEF threshold value for distinguishing whether the LV maintained the IFLSAF. A multivariate Cox proportional-hazards model revealed that the loss of IFLSAF was significantly associated with the future adverse events (HR: 7.798, 95%CI 2.174–27.969, *p* = 0.002). According to the ROC curve analysis, an LVEF ≥ 58% indicated that the LV could maintain the IFLSAF. We concluded that the loss of IFLSAF, which could reflect even slight impairment in LV contractile performance, was a reliable indicator for new-onset HF in patients with LVEF ≥ 40%. LVEF ≥ 58% could be taken as a surrogate for the IFLSAF maintenance; this threshold could be useful for risk stratification of new-onset HF in patients with preserved LVEF.

## Introduction

American and European classifications of heart failure (HF) are based on left ventricular (LV) ejection fraction (LVEF) measurements. The criteria for HF with preserved LVEF (HFpEF) are HF symptomology and LVEF ≥ 50%, which indicates LV diastolic dysfunction, despite normal LV systolic functionl [1, 2]. In addition, HF with LVEF in the range from 40 to 50% is classified as an intermediate group, or HF with a mid-range LVEF (HFmrEF). The HFmrEF class is thought to represent primarily mild decrease in LV contractile performance, combined with features of diastolic dysfunction.

LVEF represents global LV function, and it is commonly used to indicate LV contractile performance in clinical practises. However, LVEF values are influenced by several factors extrinsic to the LV, such as the preload, afterload, and heart rate, in addition to the intrinsic contractile factor and LV dilatation [3, 4]. Therefore, LVEF ≥ 50% does not accurately reflect the maintenance of normal LV contractile performance. Several previous studies performed detailed examinations of LV contractile performance in patients with LVEF ≥ 50%. Those studies demonstrated that mild impairment in LV contractile performance occurred even in patients with LVEF above 50%, or around 60% [5–10].

We hypothesized that, among patients with LVEF ≥ 50%, some might have slightly impaired LV contractile performance that could not be detected based on LVEF values. We further hypothesized that these patients might be identified with a sophisticated cardiac function parameter that could detect marginally reduced LV contractile performance. Moreover, we hypothesized that these patients also had a potential risk of future HF development. We previously reported that the inertia force of late systolic aortic flow (IFLSAF) could be calculated from LV pressure recordings with a catheter-tipped micromanometer. We demonstrated that the IFLSAF displayed a masterful ability to detect slight impairment in LV contractile performance, and this could be used as a prognostic marker of poor outcome in patients with LVEF ≥ 50% as well as those with coronary artery disease [9–11]. In the present study, with a focus on this LV contractile performance parameter of patients with normal or slightly decreased LVEF and no history of hospitalization for HF, we considered the threshold LVEF value to predict new-onset HF based on LV contractile performance in patients with preserved LVEF.

## Materials and methods

### Study population and data collection

This study retrospectively recruited 523 consecutive patients that underwent diagnostic cardiac catheterization with a catheter-tipped micromanometer to evaluate coronary artery disease, from April 2001 to December 2010. Of these patients, we enrolled 398 eligible patients that met our inclusion and exclusion criteria. The inclusion criteria included age, 20 years or older; status, no experience of hospitalization due to symptomatic HF; or no change in baseline drug therapy during the 1 month prior to enrolment. The exclusion criteria included LVEF, less than 40%; serum creatinine > 2.5 mg/dL or on haemodialysis; acute coronary syndrome requiring urgent revascularization or severe coronary artery stenosis needing early revascularization within 30 days; pacing controlled by factors other than sinus rhythm, including a pacemaker rhythm; haemodynamically significant aortic or mitral valve disease; hypertrophic cardiomyopathy; acute myocardial infarction within the past 3 months; percutaneous coronary intervention or open-heart surgery within the past 3 months; or any serious non-cardiovascular disease, including malignancy. At the time patients underwent cardiac catheterization for this study, we collected data on demographics, laboratory values, cardiac function parameters, and medications. The study endpoint was a composite of unplanned hospital admission for acute decompensated HF, and cardiovascular death. The indication for hospitalization due to acute decompensated HF was dependent on the discretion of each outpatient doctor. No one of such doctors participated in the analysis of obtained data or writing the current paper. Cardiovascular death was defined as death from congestive HF deterioration, coronary artery disease, cardiac arrest, cardiac arrhythmia, myocardial infarction, stroke, or sudden death.

### Cardiac catheterization study and IFLSAF measurement

**Fig. 1:**
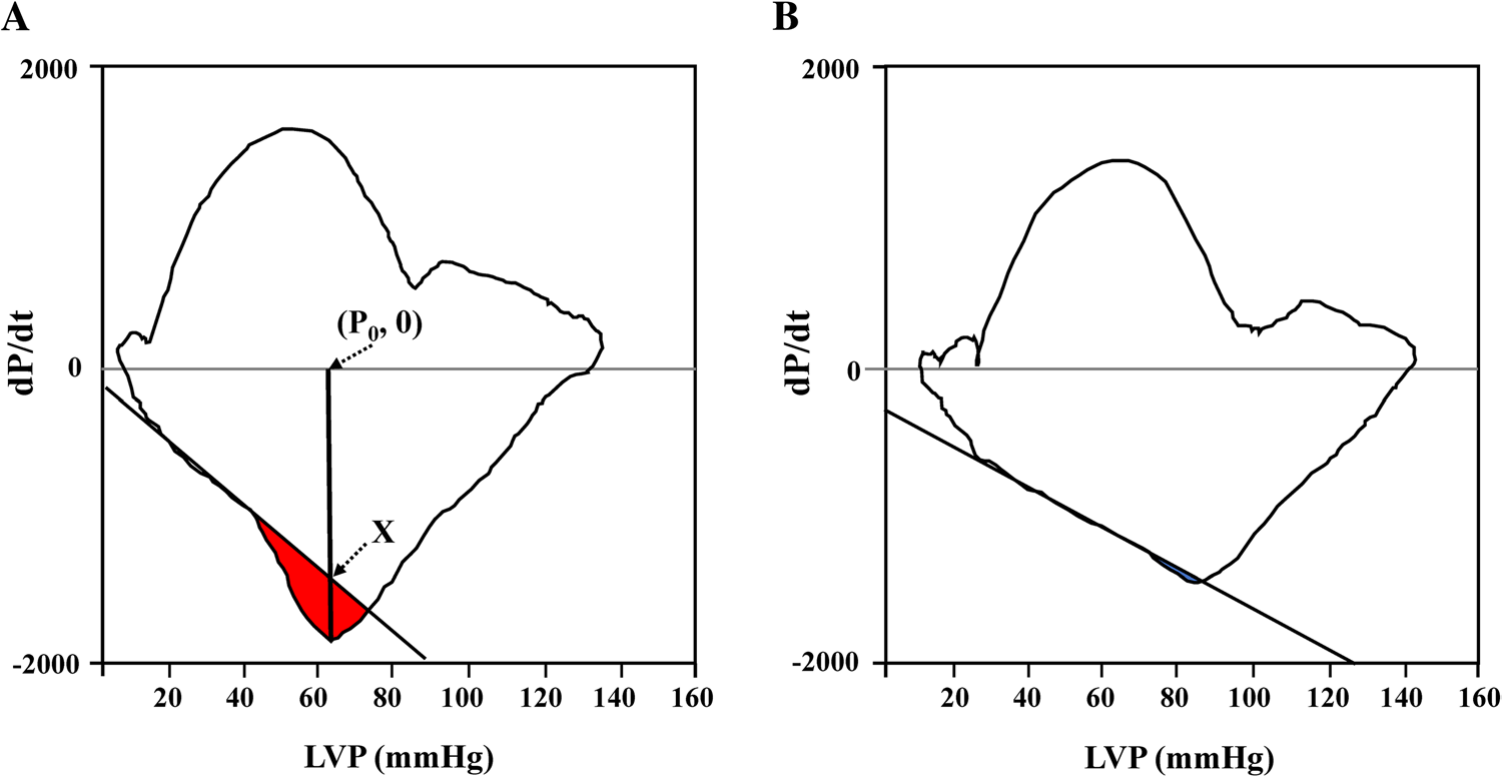
Derivation of IFLSAF from the LV pressure and dP/dt relationship. Relationship between LV pressure and the dP/dt (phase loops) show (**a**) the IFLSAF and (**b**) the loss of the IFLSAF. The IFLSAF is defined as the area in red, bounded by the phase loop and the line that best fit the section between the peak -dP/dt and the minimum LV pressure, divided by the vertical distance between (*P*_0_, 0) and point X. IFLSAF values ≥ 0.5 mmHg are considered to reflect adequate LV contractile performance

According to the procedure that we previously published [9, 10], LV pressure was measured with a catheter-tipped micromanometer (SPC-454D, Millar Instrument Co., Houston, Texas) and recorded with a polygraph system (RMC-2000 or RMC-3000, Nihon Kohden Inc., Tokyo, Japan). We also recorded LV pressure with a digital data recorder (NR-2000, Keyence, Osaka, Japan), at a sampling interval 2 ms, before injecting contrast material into the LV or coronary artery. From the recorded pressure waves, we determined the peak positive and negative first derivatives of LV pressure (± dP/dt). Furthermore, we calculated a time constant of LV pressure decay during isovolumic relaxation (*Tp*). Then, we derived the IFLSAF from the LV pressure and dP/dt relationship (phase loop plot), based on the assumptions and procedures previously described by Sugawara et al. [11]. Briefly, the *Tp* was defined as the negative inverse slope of the line with the best linear fit to the LV pressure and dP/dt relationship, in the phase between the peak -dP/dt and the minimum LV pressure from which the first several data points after peak -dP/dt and those before the minimum LV pressure were excluded. In the Sugawara method, the best linear-fit line was determined with the least squares method and expressed as: -kP + C (where k > 0; and k and C were constants to be estimated). Thus, the *Tp* was given by 1/k. Sugawara et al. hypothesized that the *Tp* might be more independent on the LV contraction phase and it might be more sensitive to the deterioration of LV relaxation than the time constant proposed by Weiss et al. [12]. Furthermore, the IFLSAF was defined as a pressure decay, augmented by the effect of the momentum of blood flowing out of the LV during late systole. The area shown in red divided by the vertical distance between (P_0_, 0) and point X is equal to the amount of pressure decay augmented by the effect of the momentum of blood (Fig. 1a). We previously defined an IFLSAF ≥ 0.5 mmHg as a significant threshold with prognostic efficacy in patients with coronary artery disease and LVEF ≥ 50% [9, 10]. In the present study, we defined maintenance of IFLSAF as IFLSAF ≥ 0.5 mmHg as in the prior study. Immediately after measuring left-sided pressure with a catheter-tipped micromanometer and right-sided pressure with a fluid-filled system, we also measured cardiac output with the thermodilution method. Then, cardiac output was used to calculate the cardiac index (cardiac output normalized by body size). In addition, we evaluated the effective arterial elastance, defined as the systolic aortic pressure divided by stroke volume. Next, we performed biplane contrast left ventriculography to determine the end-diastolic and end-systolic LV volumes and calculated the LVEF with the method described by Chapman et al. [13]. At the end of cardiac catheterization, we assessed coronary artery disease.

### Echocardiographic findings

Besides, we collected the echocardiographic findings of study patients which were measured within 2 days prior to their cardiac catheterization study. Left atrial (LA) diameter was adopted as the parameter representing LA size. LV mass was calculated by the Devereux formula [14] and normalized by the body surface area of each patient to be expressed as LV mass index (LVMI). These two parameters were also included in the cardiac function parameters of this study.

### Statistical analysis

Continuous data are presented as the mean ± SD, and categorical variables are summarized as the frequency and percentage. We used Cox proportional-hazards models and a stepwise procedure to evaluate the contributions of clinical variables, including cardiac function parameters, to the relative hazard of experiencing the composite endpoint of this study. We adopted two types of models to assess the contribution of IFLSAF: In model 1, all clinical variables were included; in model 2, the pressure parameters and brain natriuretic peptide (BNP) levels were excluded from the variables used in model 1. We used the model 2 to focus on the IFLSAF and other cardiac function parameters. A *p* value < 0.05 was considered statistically significant. We defined the day of the cardiac catheterization study as the time of patient enrolment in the study. The duration of observation in our prognosis study started at the time of enrolment and ended either at the occurrence of a terminal endpoint or at the last censoring, when the remaining patients had survived the follow-up period without any adverse events. Besides, when the study patients had no experience of concomitant HF but needed percutaneous coronary revascularization or surgical coronary artery bypass grafting, these patients were defined as censored cases at the time of coronary revascularization and the duration between the enrollment and the time of coronary interventions was adopted as the observation period for them. Furthermore, to clarify the extent of the predictive power of the IFLSAF for the endpoint, we performed a time-dependent receiver operating characteristic (ROC) curve analysis, as proposed by Heagerty et al. [15], to determine whether the IFLSAF could predict adverse events in various subsets of patients with different ranges of LVEF (lower limit varied from 40 to 70%; upper limit varied from 50 to 80%). The mean and standard error of the area under the ROC curve was calculated with 1000 datasets, created with the bootstrap resampling method, for each patient subset. In addition, we performed a regular ROC curve analysis to assess the best threshold LVEF value for discriminating whether the LV maintained the IFLSAF. Finally, we assessed correlations using Spearman's correlation coefficient by ranks between the IFLSAF and several clinical parameters, including age, hemoglobin levels, BNP levels, and cardiac pressure parameters, including the mean right atrial pressure (RAP), the mean pulmonary artery pressure (PAP), the mean pulmonary capillary wedge pressure (PCWP), and the mean aortic pressure (AP). We also assessed cardiac function parameters, including the LVEF, the LVMI, the cardiac index, the ± dP/dt, the *Tp*, and the effective arterial elastance, to clarify the clinical features of the IFLSAF.

All statistical analyses were performed with SPSS version 23.0 software (SPSS Japan Inc., Tokyo). This study was conducted in full accordance with the Declaration of Helsinki, and it received approval from the Institutional Review Boards and Ethics Committees of the Nagoya City University Graduate School of Medical Sciences, Japan.

## Results

### Patient characteristics

**Fig. 2:**
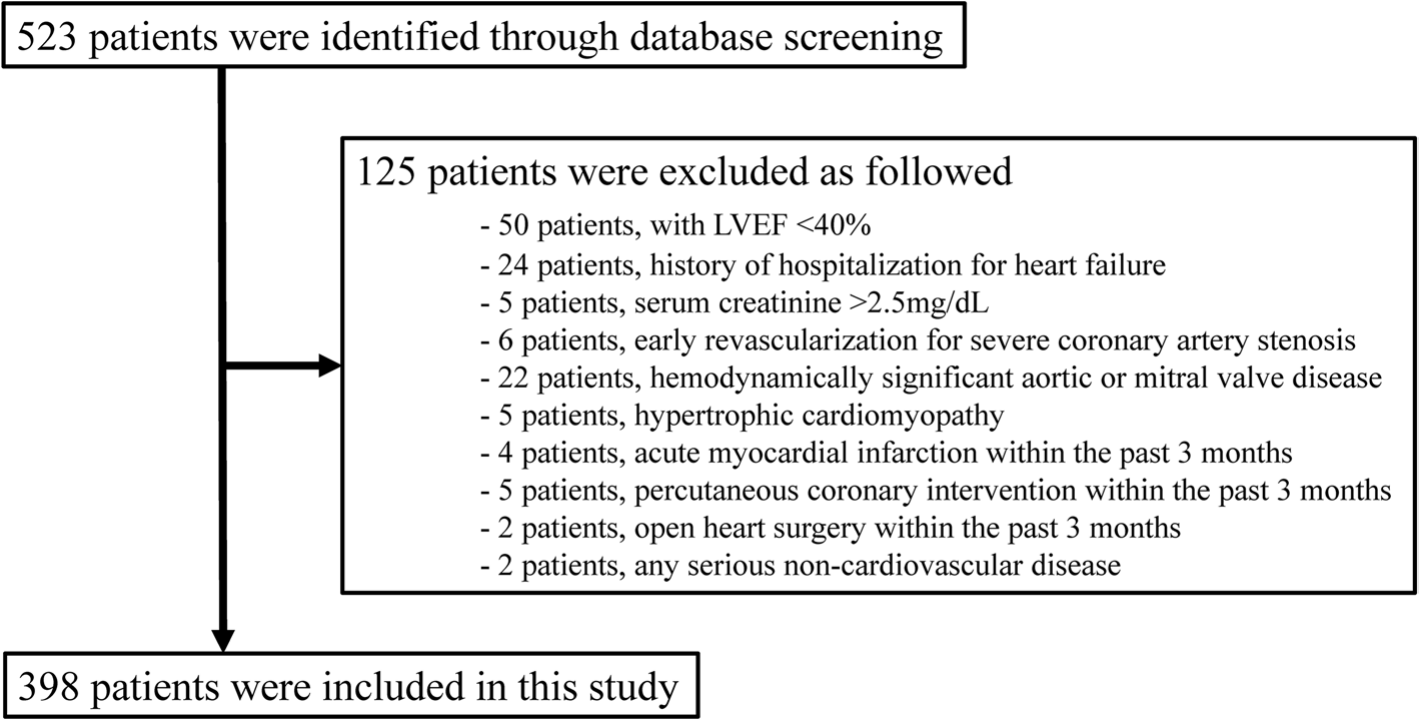
Flow-chart of patient selection. A total of 125 patients were excluded for the following reasons: with LVEF < 40% (*n* = 50); history of hospitalization for heart failure (*n* = 24); serum creatinine (sCr) > 2.5 mg/dL (*n* = 5); early revascularization for severe coronary artery stenosis (*n* = 6); hemodynamically significant aortic or mitral valve disease (*n* = 22); hypertrophic cardiomyopathy (*n* = 5); acute myocardial infarction within the past 3 months, percutaneous coronary intervention or open heart surgery within the past 3 months; (*n* = 11); any serious non-cardiovascular disease (*n* = 2)

Of the 523 consecutive patients that underwent diagnostic cardiac catheterization, 125 patients were excluded from the present study, for the reasons described in the method. (Fig. 2) Among 398 patients who were enrolled in this study, 47 patients needed percutaneous coronary revascularization and 2 patients underwent surgical coronary artery bypass grafting during the observation period. All of them did not experience concomitant HF. The characteristics of patients enrolled in this study are shown on the left side of Table 1. The mean age of all patients was 66.9 years, and 96 patients (24.1%) were female. The mean LVEF value was 66.8% and the median BNP level was in the normal range (16.6 pg/ml; interquartile range: 8.5 to 38.6 pg/ml).

**Table 1:** Patient characteristics and multivariate Cox proportional hazard regression analysis results

Characteristic	Whole Cohort (*n* = 398)	Univariate *p* value	Model 1		Model 2	
			HR (95% CI)	*P* value	HR (95% CI)	*P* value
Age, years	66.9 ± 9.2	0.741		0.953		0.670
Female	96 (24.1)	0.334		0.492		0.968
BSA, m^2^	1.67 ± 0.18	0.089		0.797		0.746
Systolic BP, mmHg	128 ± 18	0.900		0.700		0.698
Diastolic BP, mmHg	74 ± 11	0.551		0.431		0.625
Heart rate, beats/min	67 ± 12	0.926		0.359		0.405
Hemoglobin, g/dl	13.4 ± 1.5	0.060		0.759		0.377
Creatinine, mg/dl	0.83 ± 0.19	0.917		0.458		0.949
BNP, pg/ml, median (IQR)	16.6 (8.5, 38.6)			NA	NA	NA
Log BNP, pg/ml	2.90 ± 1.17	< 0.001	2.847 (1.652–4.906)	< 0.001	NA	NA
*Cardiac function*						
*Tp*, ms	81.1 ± 52.9	0.004		0.368		0.356
+ dP/dt, mmHg/s	1576.7 ± 375.6	0.174		0.427		0.895
-dP/dt, mmHg/s	-1824.1 ± 416.0	0.024		0.605		0.967
IFLAF, mmHg	3.041 ± 2.944			NA		
Loss of IFLSAF, n (%)	75 (18.8)	< 0.001		0.425	7.798 (2.174–27.969)	0.002
Cardiac index, l/min/m^2^	3.35 ± 0.67	0.805		0.625		0.823
Effective arterial elastance, mmHg/ml	1.70 ± 0.52	0.169		0.056		0.169
LVEF, %	66.8 ± 10.4	0.038		0.663		0.777
LV mass index, g/m^2^	109.5 ± 27.9	0.004	1.029 (1.002–1.056)	0.033	1.031 (1.007–1.056)	0.012
Left atrial diameter, mm	37.7 ± 7.0	0.133		0.816		0.343
*Pressure parameters*						
RAP, mmHg	4 ± 3	0.448		0.626	NA	NA
PAP, mmHg	14 ± 4	0.010		0.496	NA	NA
PCWP, mmHg	8 ± 3	0.095		0.834	NA	NA
AP, mmHg	98 ± 14	0.605		0.400	NA	NA
*Comorbidities, n (%)*						
Hypertension	230 (57.8)	0.567		0.706		0.629
Diabetes	143 (35.9)	0.391		0.864		0.347
Hyperlipidaemia	226 (56.8)	0.238		0.078		0.224
Past history of MI	168 (42.2)	0.968		0.117		0.142
*Medication, n (%)*						
ACEI and/or ARB	161 (40.5)	0.594		0.836		0.865
Beta blocker	134 (33.7)	0.748		0.509		0.309
CCB	114 (28.6)	0.227		0.145		0.181
Statin	213 (53.5)	0.286		0.068		0.080

*ACEI* angiotensin converting enzyme inhibitor, *AP* aortic pressure, *ARB* angiotensin receptor blocker, *BNP* brain natriuretic peptide, *BP* blood pressure, *BSA* body surface area, *CCB* calcium channel blocker, *dP/dt* the peak first derivative of left ventricular pressure, *IFLSAF* inertia force of late-systolic aortic flow, *IQR* interquartile range, *LVEF* left ventricular ejection fraction, *MI* myocardial infarction, *NA* not available, *PAP* pulmonary artery pressure, *PCWP* pulmonary capillary wedge pressure, *RAP* right atrial pressure, *Tp* time constant of left ventricular pressure decay during isovolumic relaxation

### Prognostic utility of IFLSAF for future adverse events

In the current study, 14 cardiovascular deaths and 17 hospitalizations for HF were documented during the follow-up period (median follow-up period: 2433.5 days; interquartile range: 1523.0 to 3387.5 days). We analysed the contribution of each parameter to the end point with two multivariate Cox proportional-hazards models (Table 1). When all parameters were included (model 1), the BNP level and the LVMI displayed a significant predictive value of adverse events (log BNP; hazard ratio (HR): 2.847, 95% confidence interval (CI) 1.652–4.906, *p* < 0.001, LVMI; HR: 1.029, 95% CI 1.002–1.056, *p* = 0.004, respectively). In model 2, which lacked the BNP level, intracardiac pressure data, and the aortic pressure value, we identified two significant predictors of adverse events; the loss of IFLSAF (HR: 7.798, 95% CI 2.174–27.969; *p* = 0.002) and the LVMI (HR: 1.031, 95% CI 1.007–1.056, *p* = 0.012).

### Prognostic power of IFLSAF and threshold LVEF value for IFLSAF maintenance

**Fig. 3:**
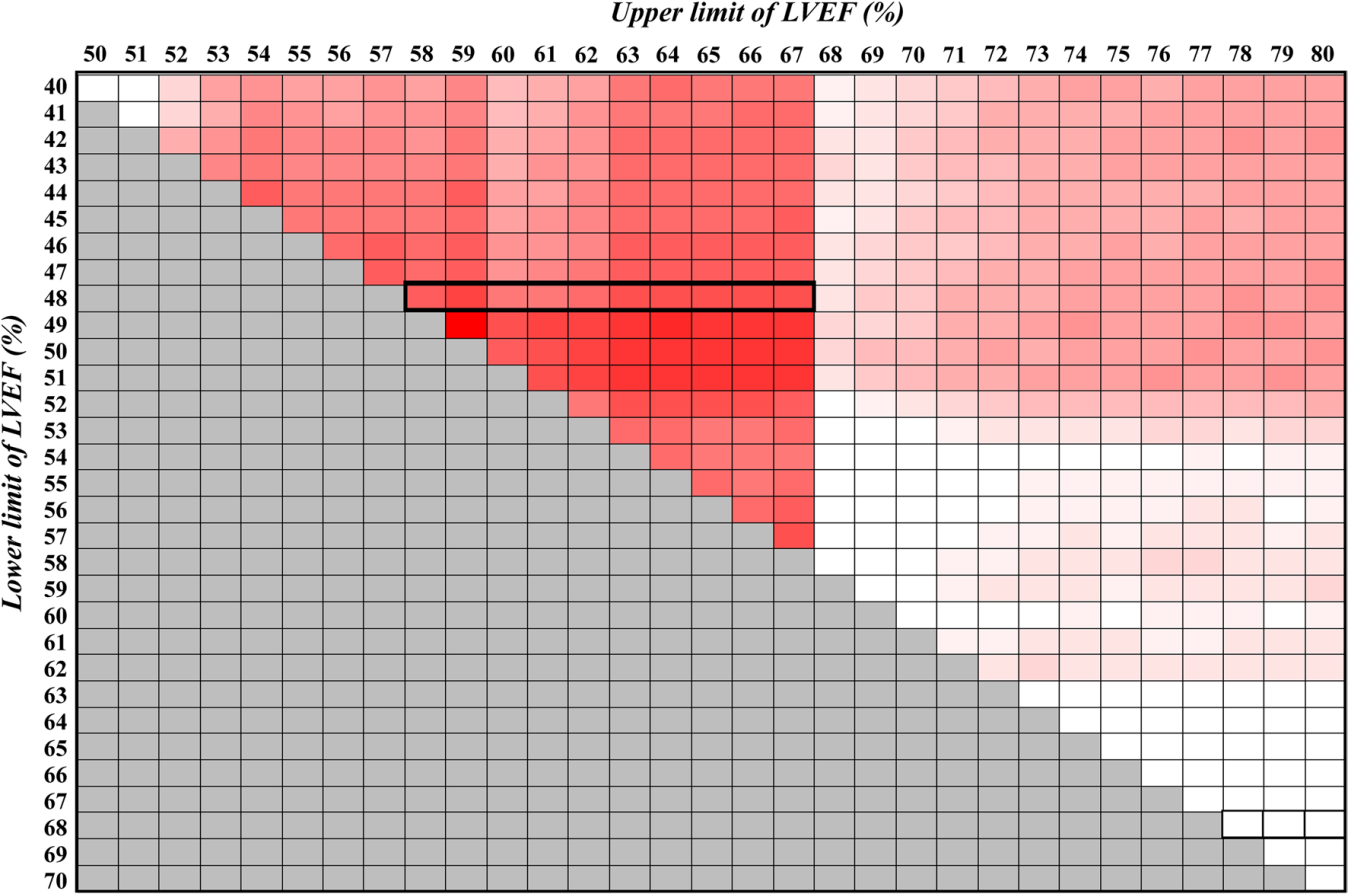
Two-dimensional heat map representing the predictive power of IFLSAF for future adverse events. Each cell represents a subset of patients with the indicated range of LVEF; the lower limit varies from 40 to 70% and the upper limit varies from 50 to 80%. Higher predictive power (AUC ≥ 0.9) is represented by a darker red colour. The maintenance of IFLSAF was a highly reliable prognostic indicator in patients with LVEF in the range from 48 to 67%

**Fig. 4:**
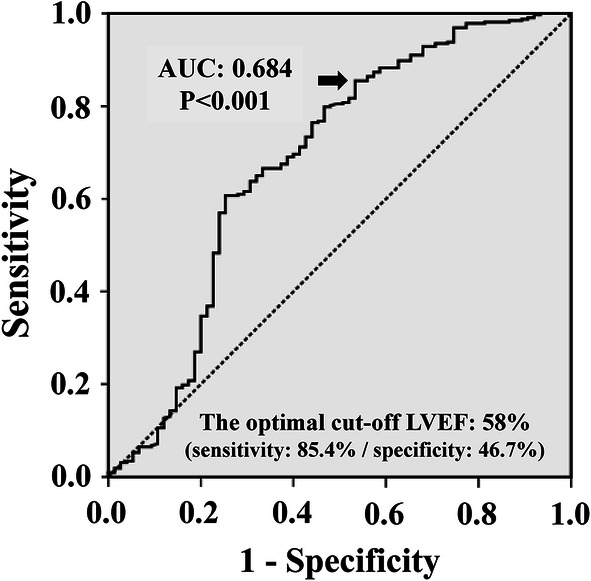
ROC curve analysis of LVEF serves as a surrogate for IFLSAF maintenance in the LV. The optimal cut-off LVEF value of 58% indicates whether the IFLSAF is maintained

Figure 3 is a two-dimensional heat map representation of the predictive power of IFLSAF for the end-point. Each cell represents a subset of patients with the indicated range of LVEF values, and the predictive power is represented with colour: higher power is represented with a darker colour. This analysis demonstrated that maintained LV contractile performance, reflected by the maintenance of IFLSAF, was a highly reliable prognostic indicator (area under the curve ≥ 0.9) in patients with LVEF in the range of 48 to 67%. Furthermore, the ROC curve analysis (Fig. 4) demonstrated that the best threshold LVEF value was 58% for discriminating whether the LV maintained the IFLSAF, with 85.4% sensitivity and 46.7% specificity.

### Correlations between IFLSAF and age, hemoglobin levels, cardiac pressure, as well as other cardiac function parameters

**Fig. 5:**
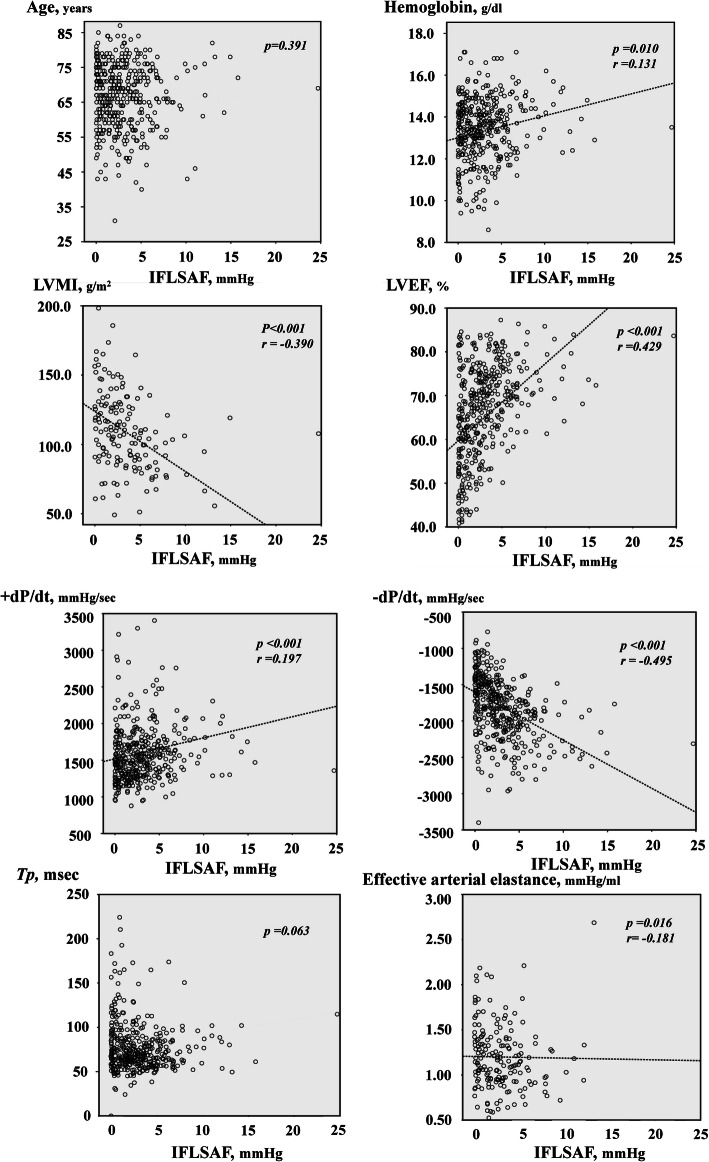
Correlations between the IFLSAF and demographic and haemodynamic variables. The LV mass index, LVEF, peak -dP/dt, and BNP levels had significant and relatively high correlations with the IFLSAF. In contrast, hemoglobin levels, peak + dP/dt, and effective arterial elastance showed significant, but relatively poor correlations with the IFLSAF

We performed correlation analyses to examine the association between the IFLSAF and age, hemoglobin levels, BNP levels, the effective arterial elastance, the cardiac pressure parameters, as well as other cardiac function parameters shown in Table 1 (Fig. 5). We found that the IFLSAF was strongly correlated with the LVMI, the LVEF level, the peak -dP/dt, and the BNP level (LVMI, *r* =  − 0.390, *p* < 0.001; LVEF: *r* = 0.429, *p* < 0.001; -dP/dt: *r* =  − 0.495, *p* < 0.001; and log BNP: *r* =  − 0.358, *p* < 0.001, respectively). In contrast, the hemoglobin level, the peak + dP/dt, and the effective arterial elastance showed significant, but relatively poor correlations with the IFLSAF (hemoglobin: *r* = 0.131, *p* = 0.010; + dP/dt: *r* = 0.197, *p* < 0.001; effective arterial elastance: *r* =  − 0.181, *p* = 0.016, respectively). We found no significant correlation between the IFLSAF and the cardiac index, *Tp*, or the cardiac pressure parameters.

## Discussion

The present study had four major findings. First, we found that mild decrease in LV contractile performance, which was reflected by a loss of the IFLSAF, was an important predictor of new-onset HF in patients with normal or slightly decreased LVEF (≥ 40%). Second, we found that, among patients with LVEF that ranged from 48 to 67%, a loss of the IFLSAF could strongly predict poor prognosis. Third, we demonstrated that the LVEF cut-off value of 58% could serve as a surrogate for determining whether the LV maintained the IFLSAF in patients with LVEF ≥ 40%. Finally, the IFLSAF was correlated with the LVMI, the LVEF, the peak -dP/dt, and the BNP level.

The LVEF has been widely used as a parameter of LV contractile performance for predicting the development of heart failure and cardiovascular death in the general population, in patients with asymptomatic reductions in LVEF, and in patients with symptomatic HF [16, 17]. In addition, previous studies demonstrated that LVEF was related to differences in patient demographics, comorbid conditions, and response to therapies [18]. Therefore, LVEF is considered important in classifications of patients with HF. However, it has been challenging to set a range of LVEF that represented no reduction in LV contractile performance. Several detailed examinations of LV contractile performance have observed slight impairment in the performance, even in patients with LVEF ≥ 50%, which is typically classified as a preserved LVEF [5–10]. Similarly, in the current study, 15.3% of patients with LVEF ≥ 50% exhibited decreased LV contractile performance, which was reflected by a loss of the IFLSAF (18.8% in the whole study patients). Previous studies have used echocardiography to investigate the prevalence of decreased LV contractile performance in patients with LVEF ≥ 50%, patients with HFpEF risk factors, such as hypertension and diabetes mellitus, and patients with HFpEF [7, 19–21]. According to the study by Shah et al. [7], an abnormality in LV contractile performance could be detected by a reduction in the longitudinal strain of the LV. This LV strain reduction was shown to have prognostic utility in patients with HFpEF in the TOPCAT trial (Treatment of Preserved Cardiac Function Heart Failure with an Aldosterone Antagonist) [22]. Their findings suggested that decreased LV contractile performance might underlie the pathophysiology in some patients with HFpEF. Consistently, in the present study, we also demonstrated that decreased LV contractile performance, reflected by a loss of the IFLSAF, was an independent predictor of new-onset HF and cardiovascular death in patients with LVEF ≥ 40%. Furthermore, we identified the range of LVEF over which the IFLSAF had prognostic power, based on a time-dependent ROC curve analysis. We found that, among patients with LVEF between 48 and 67%, mild impairment in LV contractile performance, indicated by a loss of the IFLSAF, could lead to future HF development.

HFmrEF was recently recognized as a new general category of HF, different from both HF with reduced LVEF (HFrEF) and HFpEF. However, the pathophysiological mechanisms underlying HFmrEF and the threshold LVEF values for differentiating among the three general categories of HF have not been elucidated. Our main findings suggested that mildly decreased LV contractile performance was associated with HF development in patients with LVEF ≥ 40%. Our findings also suggested that the extent of pathophysiological effects of the decrease in LV contractile performance on HF occurrence could be shown in patients with the LVEF from 48 to 67% who had not experienced hospitalization for HF previously. When patients have mild decrease in LV contractile performance that could lead to HF development, the condition might be classified as HFmrEF. In addition, when the classification of a normal LVEF is defined as no reduction in LV contractile performance, based on the maintenance of IFLSAF, the patients who have LVEF of around 67% or greater are considered with normal contractile performance.

Currently, the only promising medical treatments for patients with HF are for patients with decreased LV contractile performance (LVEF < 35 ~ 40%). When we reconsider the HF classification from the viewpoint of responses to medical treatments for HF, the classifications may not be based on the already established LVEF value, but on LV contractile performance, irrespective of whether or not the LVEF is ≥ 40%. A sub-analysis, reported by Solomon et al. in the TOPCAT trial [23], demonstrated that the estimated benefits of mineralocorticoid receptor antagonist were stronger in patients with LVEF at the lowest end of the spectrum than in patients with LVEF at the highest end of the spectrum in patients with LVEF ≥ 45%. This finding demonstrated the importance of differentiating patients with HF based on LV contractile performance, due to different responses to medical treatments, even among patients with LVEF ≥ 40%. Although, to date, no convincing medical treatment has been shown to reduce morbidity or mortality in patients with LVEF ≥ 40%, we speculate that by identifying patients with HFmrEF based on LV contractile performance, we could treat a larger proportion of HF patients with drug therapy regimens that were designed for patients with HFrEF.

In this study, we performed a ROC curve analysis to assess the best threshold LVEF value for determining whether the LV was with the IFLSAF. We demonstrated that LVEF values < 58% could predict a loss of the IFLSAF with high sensitivity (85.4%) in patients with LVEF ≥ 40%. This finding indicated that patients with LVEF < 58% had a potential risk of decrease in LV contractile performance that could lead to the development of HF in the future, despite a LVEF > 40%. Therefore, taking decrease in LV contractile performance as a risk factor for new-onset HF, we propose that the upper LVEF cut-off value between HFmrEF and HFpEF should be around 58%. Several previous reports have supported the notion that the LVEF threshold for defining HFpEF should be raised above the 50% threshold commonly used. Some community-based cohort studies demonstrated that the persons with LVEF 55 to 60% had greater risk for morbidity and mortality compared to those with LVEF 60% [24–26]. In addition, patients with HFpEF that had LVEF < 55% were reported to be significantly associated with a risk of the LVEF declining to below 50%, which means that the patients would shift to more severe decrease in LV contractile performance [27]. Furthermore, when study patients were classified based on another threshold LVEF value a bit higher than 55%, the clinical features of HFpEF were heterogeneous. According to a sub-analysis in the I-PRESERVE Study (Irbesartan in Heart Failure with Preserved Ejection Fraction) [28], the prognostic impact of LVEF on HFpEF was significantly different when LVEF was below 60% compared to when LVEF was 60% or greater. Additionally, in a sub-analysis of the J-MELODIC trial (Japanese Multicenter Evaluation of Long- vs. short-acting Diuretics In Congestive heart failure), we previously demonstrated that HFpEF was heterogeneous based on the prognostic utility of BNP levels in the LVEF ranges (40–60% and ≥ 60%) [29]. Furthermore, Solomon et al. also demonstrated the effect of angiotensin receptor-neprilysin inhibition (ARNI) in patients with symptomatic heart failure and LVEF ≥ 45% in the PARAGON-HF trial (The Prospective Comparison of ARNI with Angiotensin-receptor blockers Global Outcomes in Heart Failure with Preserved Ejection Fraction) [30]. Although ARNI did not reduce the rate of total hospitalizations for HF and death from cardiovascular causes among whole study patients, for the patients with relatively lower LVEF (LVEF ≤ 57%), ARNI significantly reduced morbidity and mortality. The PARAGON-HF trial did demonstrate the efficacy of ARNI in patients with the very similar LVEF range where a decrease in LV contractile performance was observed as the loss of the IFLSAF in the current study. These findings support that a LVEF value < 58% indicates mild decrease in LV contractile performance in patients with LVEF ≥ 40% from the viewpoint of drug effects.

This study had several limitations. First, the study design was retrospective, and we analysed data from a single institution. We recruited study patients from those who underwent diagnostic cardiac catheterization for the evaluation of coronary artery disease concomitantly with a sophisticated measurement of LV pressure with a catheter-tipped micromanometer. It may be associated with lower proportion of female in the study patients. Second, all patients had some HFpEF risk factors, such as hypertension and diabetes mellitus, but no history of hospitalization for HF. Because this study focused on the potential risk of the impairment of LV contractile performance in patients with LVEF ≥ 40% for new-onset HF, the patients with a history of hospitalization for HF were excluded. When we discuss a threshold LVEF value for distinguishing between HFpEF and HFmrEF, we should give consideration to the distinction in the mechanism underlaying HF between new-onset and recurrence HF in patients with LVEF ≥ 40%. In addition, all our patients were in sinus rhythm. Thus, a future prospective study is needed to strengthen our conclusions, with a larger study cohort that includes more patients with a history of hospitalization for HF and atrial fibrillation. Third, we focused on the IFLSAF as a marker of LV contractile performance. Although, compared to LVEF, the IFLSAF could detect small reductions in LV contractile performance, the IFLSAF measurement was somewhat complicated and invasive, because it required LV pressure recordings with a catheter-tipped micromanometer. This procedure is not practical for the bed-side management of patients with HF. Consequently, a more practical parameter for representing LV contractile performance, which could be measured by non-invasive approach such as echocardiography or cardiac magnetic resonance imaging, might be necessary. Finally, we did not address changes in LV contractile performance over the course of HF, nor did we investigate the association between changes in LV contractile performance and prognosis.

In conclusion, we demonstrated that mild reduction in LV contractile performance, indicated by a loss of the IFLSAF, was one of the prognostic indicators for new-onset HF in patients with LVEF ≥ 40%. We showed that an LVEF ≥ 58% could be taken as a surrogate for the IFLSAF maintenance. Moreover, medical treatments that are efficacious for HFrEF might be applicable for the patients with their LVEF < 58%.
